# Late TNFα blockade is associated with systemic immune rewiring without disease control in established experimental autoimmune hepatitis

**DOI:** 10.1016/j.jtauto.2026.100396

**Published:** 2026-08-25

**Authors:** Laura Elisa Buitrago-Molina, Zexuan Shi, Ziting Zhao, Heiner Wedemeyer, Fatih Noyan, Matthias Hardtke-Wolenski

**Affiliations:** a Dept. of Gastroenterology, Hepatology, Infectious Diseases and Endocrinology, Hannover Medical School, Germany; b Inst. of Medical Microbiology, University Hospital Essen, University Duisburg-Essen, Essen, Germany

**Keywords:** Autoimmune hepatitis, TNFα, Infliximab, Experimental autoimmune hepatitis, Immune rewiring, CCL2, Regulatory T cells

## Abstract

Recent human studies have identified TNFα as a therapeutically targetable pathway in autoimmune hepatitis (AIH), but the consequences of TNFα blockade may depend on treatment timing and disease context. Whether TNFα neutralization can restore disease control once autoimmune liver inflammation is already established remains unclear.

Here, we investigated late TNFα blockade in experimental autoimmune hepatitis (emAIH). Anti-TNFα treatment was initiated after disease establishment, and mice were analyzed at early and late post-treatment time points by integrating serum biochemistry, hepatic inflammatory infiltrate quantification, liver gene expression, flow cytometry, serum proteomics and regulatory immune readouts.

Late TNFα blockade did not improve serum ALT or AST levels and did not reduce hepatic inflammatory infiltrate area compared with isotype-treated controls. Nevertheless, anti-TNFα treatment was associated with hepatic transcriptional remodeling, including reduced Acta2 expression and directional changes in Ccl2 and Fgf21 at the early time point. At the later time point, anti-TNFα treatment was associated with increased the frequencies of IFN-γ- and TNFα-producing splenic CD4^+^ T cells, whereas intrahepatic cytokine-producing T-cell frequencies remained largely unchanged. Serum proteomics identified late systemic inflammatory remodeling, including increased CCL2 abundance. In contrast, hepatic Foxp3 expression and absolute hepatic Treg numbers were not increased.

Together, these findings indicate that late TNFα blockade in established emAIH remains biologically active but is uncoupled from biochemical improvement and reduced hepatic inflammatory infiltration. The data complement recent human infliximab studies by suggesting that timing and disease context may shape the consequences of TNFα-targeted therapy in AIH.

## 1 Introduction

Autoimmune hepatitis (AIH) is a chronic immune-mediated liver disease that can progress to fibrosis and cirrhosis and is associated with adverse long-term outcomes when biochemical control is incomplete [[1], [2], [3]]. Although most patients respond to corticosteroids with or without azathioprine, a clinically relevant subgroup remains intolerant, incompletely responsive, or refractory, prompting the use of alternative second-line and rescue strategies [[1], [2], [3]]. In this clinically challenging setting, second-line thiopurines, B-cell–directed approaches, and biologics are increasingly considered [[4], [5], [6]], although mechanistic guidance for therapeutic selection remains limited despite emerging real-world data on infliximab rescue [7].

Recent human data have renewed interest in tumor necrosis factor alpha (TNFα) as a therapeutically relevant pathway in AIH. Xu et al. identified TNF as a key inflammatory node in newly diagnosed AIH using integrative multi-omics and further demonstrated clinical efficacy of infliximab as steroid-free induction therapy in a phase IIa study [8]. In parallel, multicenter real-world data by Efe et al. showed that infliximab can induce biochemical remission in a subset of difficult-to-treat AIH patients but also highlighted heterogeneous responses and clinically relevant infectious complications [7]. Together, these human studies identify TNFα as a clinically relevant and therapeutically targetable pathway in AIH, while underscoring that the consequences of TNFα blockade may depend on treatment timing, disease context, and patient selection.

This distinction is biologically plausible. TNFα is a pleiotropic cytokine involved in inflammatory signaling, tissue remodeling, and context-dependent immune regulation [9]. In addition to its pro-inflammatory effects, TNFα contributes to T-cell homeostasis and can influence regulatory T-cell stability, at least in part through TNFR2-dependent mechanisms [10,11]. Human studies in AIH further indicate that the regulatory compartment is heterogeneous, with reports of altered Treg function, treatment-associated depletion of intrahepatic Tregs, and preserved or even inflammation-associated accumulation of FOXP3^+^ cells in other cohorts [[12], [13], [14]]. Experimental AIH models similarly indicate that chronic disease states are maintained by compensatory immune networks rather than by a single dominant pathway [15,16].

To address this timing- and context-dependent question, we examined late TNFα neutralization in established experimental murine autoimmune hepatitis (emAIH) [15,16]. We did not aim to reproduce the clinical setting of newly diagnosed AIH treated with infliximab induction therapy. Instead, we asked whether TNFα blockade initiated after disease establishment can still improve biochemical activity or reduce hepatic inflammatory infiltration, or whether it primarily reshapes hepatic molecular programs and systemic immune responses. By integrating serum biochemistry, quantification of hepatic inflammatory infiltrates, hepatic gene expression, flow cytometry, and serum proteomics, we aim to distinguish measurable disease control from treatment-associated molecular and compartment-specific immune remodeling in established autoimmune liver inflammation.

## 2 Material and methods

### 2.1 Mice and animal ethics

Male NOD/Ltj mice aged 6–8 weeks were used for all experiments. NOD/Ltj mice were selected because the AdFTCD emAIH model was previously developed and longitudinally characterized in this autoimmune-prone background [15,16]. Previous model-development studies demonstrated that genetic susceptibility critically determines progression to chronic autoimmune liver inflammation and identified NOD/Ltj mice as the susceptible background for chronic AdFTCD-induced emAIH [15,16]. The present study therefore used this validated model to test a therapeutic intervention rather than to compare strain susceptibility. Male mice were used to reduce the risk that spontaneous autoimmune diabetes, which can occur in the NOD background, confounds the assessment of autoimmune liver inflammation. Blood glucose was measured every second day throughout the experiment. Animals developing diabetes, defined as blood glucose levels >200 mg/dL, or reaching predefined endpoint criteria were removed from further experimental follow-up according to the approved animal protocol. These criteria were prospectively defined and applied irrespective of treatment response or experimental outcome.

Mice were maintained under specific pathogen-free conditions at the central animal facility of Hannover Medical School with ad libitum access to food and water. Animal health status was monitored regularly by trained personnel. All procedures were approved by the Animal Welfare Commission of Hannover Medical School and the Lower Saxony State Office for Consumer Protection and Food Safety, Oldenburg, Germany, under approval number 16/2116, including subsequent amendments, and 23-00531. At the experimental endpoint, mice were euthanized under deep CO_2_ anesthesia followed by cervical dislocation, as specified in the approved protocol. The manuscript was prepared in accordance with the ARRIVE guidelines.

### 2.2 Induction of experimental autoimmune hepatitis and anti-TNFα treatment

Experimental autoimmune hepatitis was induced by intravenous administration of adenovirus encoding formiminotransferase cyclodeaminase (AdFTCD; 2 × 10^9^ infectious particles in PBS), as previously described [15,16]. Week 12 was deliberately selected as the start of therapeutic intervention based on the previously established kinetics of this model. Initial AdFTCD administration causes a transient adenovirus-associated hepatitis followed by chronic autoimmune liver inflammation; by week 12, NOD/Ltj mice display established autoimmune hepatitis with persistent inflammatory infiltrates and increased inflammatory infiltrate size [15,16]. Quantitative inflammatory infiltrate size is an established objective histological readout in the characterization of this model [15,16]. Thus, week 12 represents an established chronic disease time point rather than the induction phase. At week 12, mice were allocated to receive either a neutralizing anti-mouse TNFα antibody or the corresponding isotype control. Individual pretreatment liver histology was not performed because quantitative histological assessment requires terminal tissue collection and would preclude subsequent longitudinal treatment of the same animal.

Anti-TNFα treatment was performed using anti-mouse TNFα antibody clone XT3.11 at a dose of 500 μg per mouse twice weekly by intraperitoneal injection. Control mice received the matched isotype control antibody according to the same schedule. Two endpoint cohorts were analyzed at weeks 18 and 24 after AdFTCD administration.

Final group sizes differed between individual analyses because NOD/Ltj mice may develop diabetes or reach predefined humane endpoint criteria during longitudinal experiments, and because sample availability varied between downstream assays. No post hoc replacement of animals was performed. Exact numbers of animals included in each analysis are reported in the Results section and figure legends.

### 2.3 Adenovirus construction

The generation of AdFTCD has been described previously [15,16]. Briefly, FTCD was amplified from human liver cDNA, and the sequence was verified by sequencing both DNA strands. The construct was cloned into the adenoviral transfer vector pShuttle-CMV. By homologous recombination, this shuttle vector was recombined with pAdEasy-1, which carries deletions in the E1 and E3 regions. The resulting adenoviral genome was amplified in HEK293 packaging cells, which complement the essential adenoviral regions. Recombinant adenovirus was purified by cesium chloride gradient centrifugation, and adenoviral stocks were quantified using an Adeno-X Rapid Titer Kit.

### 2.4 Sample collection

At weeks 18 or 24 after AdFTCD administration, blood was collected via the retro-orbital route for serum preparation. Serum was used for biochemical analysis and Olink proteomic profiling. Liver and spleen were harvested for histology, RNA extraction, and immune-cell profiling. Liver tissue for histology was fixed in formalin and embedded in paraffin. Liver tissue for RNA analysis was snap-frozen and stored at −80°C until further processing. Liver and spleen samples for flow cytometry were processed immediately into single-cell suspensions.

### 2.5 Serum biochemistry

Serum aspartate aminotransferase (AST) and alanine aminotransferase (ALT) activities were determined by photometric enzymatic activity assays using an Olympus AU400 Chemistry Analyzer as described previously [15,16]. Results are reported as U/L.

### 2.6 Histology and quantification of inflammatory infiltrates

Formalin-fixed, paraffin-embedded liver tissue was sectioned at 5 μm and stained with hematoxylin and eosin as described previously [15,16]. Representative images were acquired using an AxioImager microscope and AxioVision software.

Histological assessment was performed in a blinded manner. Inflammatory liver pathology was quantified by measuring the area occupied by inflammatory infiltrates in hematoxylin-and-eosin-stained liver sections using AxioVision software, as described previously. For each animal, two liver sections and ten microscopic fields were analyzed. Individual inflammatory foci were not counted separately; instead, the total inflammatory infiltrate area within the analyzed fields was quantified. The mean infiltrate area per animal was used for statistical comparison.

### 2.7 RNA isolation and quantitative real-time PCR

Total RNA was isolated from frozen liver tissue using the RNeasy Mini Kit according to the manufacturer's instructions and quantified using a NanoDrop 1000 spectrophotometer. Two micrograms of RNA were reverse-transcribed into cDNA using the Transcriptor High Fidelity cDNA Synthesis Kit.

For quantitative real-time PCR, cDNA was preamplified using Fluidigm PreAmp Master Mix and commercial TaqMan for 14 pre-amplification cycles assays according to the manufacturers’ instructions. Quantitative RT-PCR was performed in a 48.48 Dynamic Array integrated fluidic circuit using preamplified samples. IFC priming and loading were performed on a Juno instrument, and RT-PCR data were generated using a BioMark HD system.

Hepatic expression of Acta2, Ccl2, Fgf21, Foxp3, Tnf and additional indicated target genes was quantified as described previously [17,18]. Ct values were normalized by subtracting the mean Ct of the housekeeping genes Gapdh and Actb from the Ct of the gene of interest. Relative expression was calculated and normalized to the corresponding isotype-treated control group unless stated otherwise.

### 2.8 Isolation of liver and spleen immune cells

Liver and spleen tissue were mechanically dissociated. Intrahepatic lymphocytes were isolated using a 40%/70% Percoll gradient. Red blood cells in spleen single-cell suspensions were lysed before staining. Cells were washed, counted, and processed for flow cytometric analysis as described previously [15,16].

### 2.9 Flow cytometry and intracellular cytokine staining

For phenotypic and regulatory T-cell analyses, liver- and spleen-derived single-cell suspensions were stained with combinations of commercially available antibodies against CD45, CD3, CD4, CD8, CD25, CD62L, Ki67, Foxp3 and additional indicated markers. Regulatory T cells were defined as CD4^+^CD25^+^Foxp3^+^ cells unless otherwise stated.

For intracellular cytokine analysis, cells were stimulated ex vivo with PMA/ionomycin in the presence of protein transport inhibitors before fixation and permeabilization. Intracellular staining was performed for IFN-γ and TNFα. Cells were gated sequentially on singlets, viable lymphocytes, CD45^+^ leukocytes, CD3^+^ T cells, and CD4^+^ or CD8^+^ T-cell subsets. Cytokine-positive and Foxp3^+^ gates were defined using unstimulated, fluorescence-minus-one and/or internal negative controls where appropriate.

All data were acquired on an LSRII SORP flow cytometer interfaced with FACSDiva software and analyzed using FlowJo version 10.10, as described previously [17,18].

### 2.10 Olink serum proteomics

Serum inflammatory protein profiling was performed using the Olink Mouse Inflammation panel according to the manufacturer's instructions as described previously [17,19]. The assay is based on proximity extension assay technology and enables simultaneous quantification of inflammatory and immune-related serum proteins.

Briefly, pairs of oligonucleotide-labelled antibody probes bind to their target proteins. When both probes are brought into proximity, the oligonucleotides hybridize and are extended by DNA polymerase, generating a protein-specific PCR target sequence. The resulting DNA sequence was detected and quantified using a BioMark HD microfluidic real-time PCR instrument. Data were quality-controlled and normalized using the standard Olink workflow, including internal extension and interplate controls. No additional user-defined background subtraction or background correction was applied before downstream statistical analysis. Protein abundance is reported as normalized protein expression (NPX) values on a log2 scale, with higher NPX values corresponding to higher protein abundance.

Proteomic analyses were performed separately for week 18 and week 24. Differential abundance between anti-TNF-α-treated and isotype-treated mice was assessed for each time point. P values were adjusted for multiple testing using the Benjamini–Hochberg false-discovery-rate procedure.

### 2.11 Statistical analysis

Data are shown as individual animals with mean ± SD unless stated otherwise. Statistical analyses were performed using GraphPad Prism and Qlucore Omics Explorer where appropriate. Comparisons between two groups were performed using the two-tailed Mann–Whitney *U* test. Exact p values are shown in the figures where applicable. A p value < 0.05 was considered statistically significant for conventional group comparisons.

For Olink proteomic analyses, p values were adjusted using the Benjamini–Hochberg false-discovery-rate procedure. Adjusted q values < 0.05 were considered statistically significant for proteomic analyses. Given the small cohort size, proteomic findings were interpreted as exploratory unless supported by concordant cellular or transcriptional data.

Final group sizes reflect the number of animals that completed the approved longitudinal protocol without diabetes development, defined as blood glucose levels >200 mg/dL, or predefined humane endpoint criteria, and for which sufficient sample material was available for the respective downstream assay. Exact n values are provided in the corresponding figure legends.

## 3 Results

### 3.1 Late TNFα blockade does not improve biochemical activity or hepatic inflammatory infiltration in established emAIH

To determine whether TNFα blockade attenuates established autoimmune liver inflammation, emAIH was induced in male NOD/Ltj mice by intravenous AdFTCD administration, and treatment with anti-TNFα antibody or isotype control was initiated at week 12, the established chronic disease time point defined by previous longitudinal characterization of the model [15,16]. Two endpoint cohorts were analyzed at weeks 18 and 24 after AdFTCD administration (Fig. 1A).

**Fig. 1:**
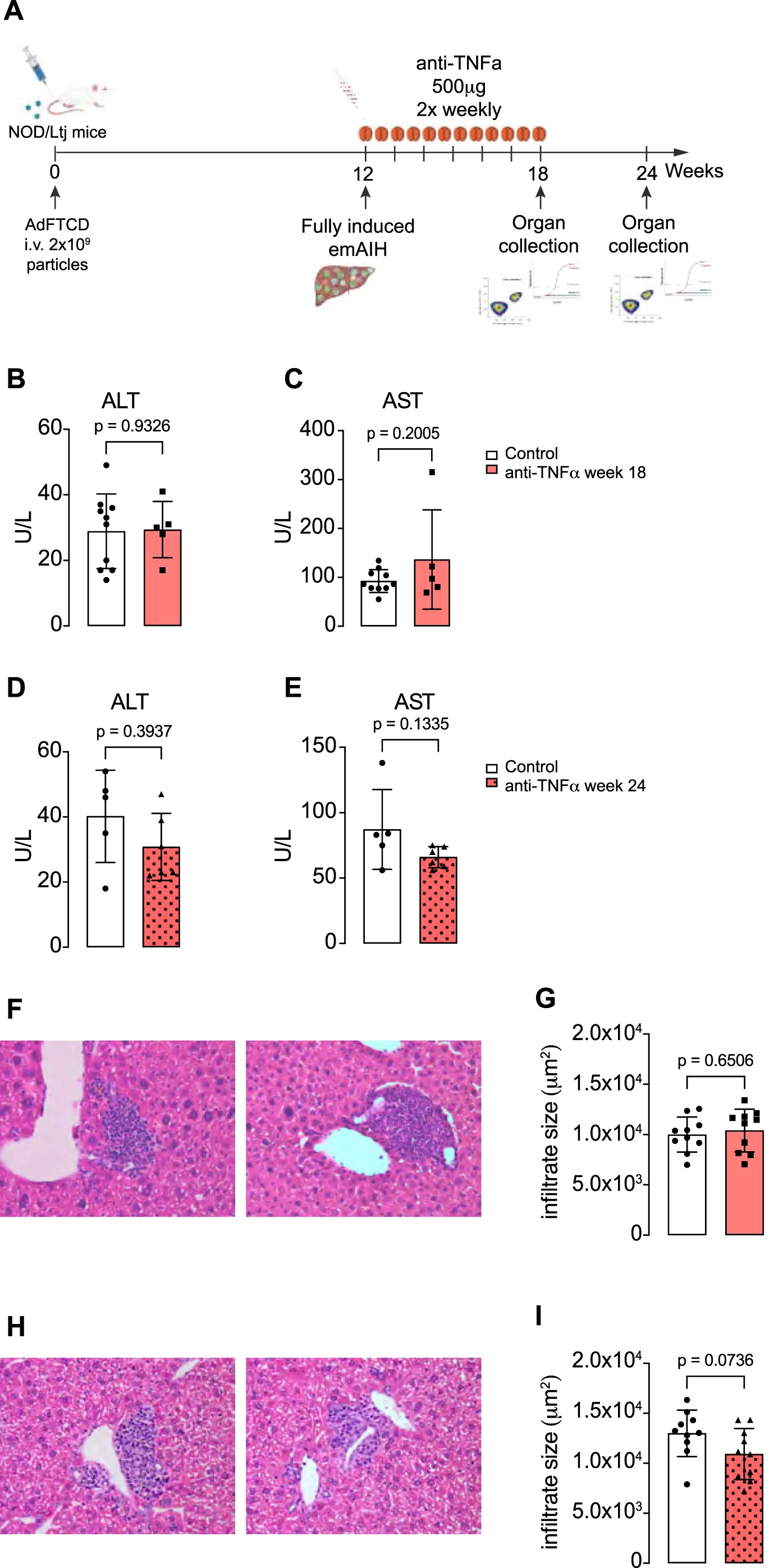
**Late TNFα blockade does not improve biochemical activity or hepatic inflammatory infiltration in established experimental autoimmune hepatitis.** (A) Experimental design. Experimental autoimmune hepatitis was induced in male NOD/Ltj mice by intravenous administration of AdFTCD. After disease establishment at week 12, mice received *anti*-TNFα antibody or isotype control twice weekly until analysis at week 18 or week 24. (B–C) Serum alanine aminotransferase (ALT) and aspartate aminotransferase (AST) levels at week 18. (D–E) Serum ALT and AST levels at week 24. (F) Representative hematoxylin-and-eosin-stained liver sections at week 18. (G) Quantification of hepatic inflammatory infiltrate area at week 18. (H) Representative hematoxylin-and-eosin-stained liver sections at week 24. (I) Quantification of hepatic inflammatory infiltrate area at week 24. For histological quantification, two liver sections and ten microscopic fields per animal were analyzed. Individual inflammatory foci were not counted separately; the total inflammatory infiltrate area within the analyzed fields was quantified and the mean value per animal was used for statistical comparison. Each dot represents one animal; horizontal bars indicate the median. Statistics: Mann–Whitney *U* test, two-tailed. Exact p values are shown.

Serum alanine aminotransferase (ALT) and aspartate aminotransferase (AST) levels were comparable between anti-TNFα-treated and isotype-treated mice at week 18 (Fig. 1B and C). Likewise, anti-TNFα treatment did not reduce ALT or AST levels at week 24 (Fig. 1D and E). Thus, late TNFα blockade did not improve biochemical disease activity under the conditions tested.

Histological analysis supported this conclusion at the tissue level. Representative hematoxylin-and-eosin-stained liver sections showed persistent inflammatory infiltrates in both anti-TNFα-treated and isotype-treated mice at week 18 and week 24 (Fig. 1F–H). Quantification of hepatic inflammatory infiltrate area did not show a significant reduction after TNFα blockade at either time point (Fig. 1G–I). Together, these biochemical and histological data indicate that TNFα neutralization initiated after disease establishment does not confer measurable biochemical improvement or reduce hepatic inflammatory infiltration in established emAIH.

### 3.2 TNFα blockade induces hepatic transcriptional remodeling despite absent disease improvement

Although late TNFα blockade did not improve serum transaminases or hepatic inflammatory infiltration, it was associated with measurable hepatic transcriptional changes at week 18. Hepatic Acta2 expression was significantly reduced in anti-TNFα-treated mice compared with isotype-treated controls (Fig. 2A), indicating modulation of a tissue-remodeling-associated gene. In addition, Ccl2 and Fgf21 showed directional changes without reaching statistical significance (Fig. 2B and C). These findings suggest that late TNFα blockade remains biologically active at the hepatic molecular level, but that these transcriptional effects are insufficient to translate into biochemical improvement or reduced inflammatory infiltration.

**Fig. 2:**
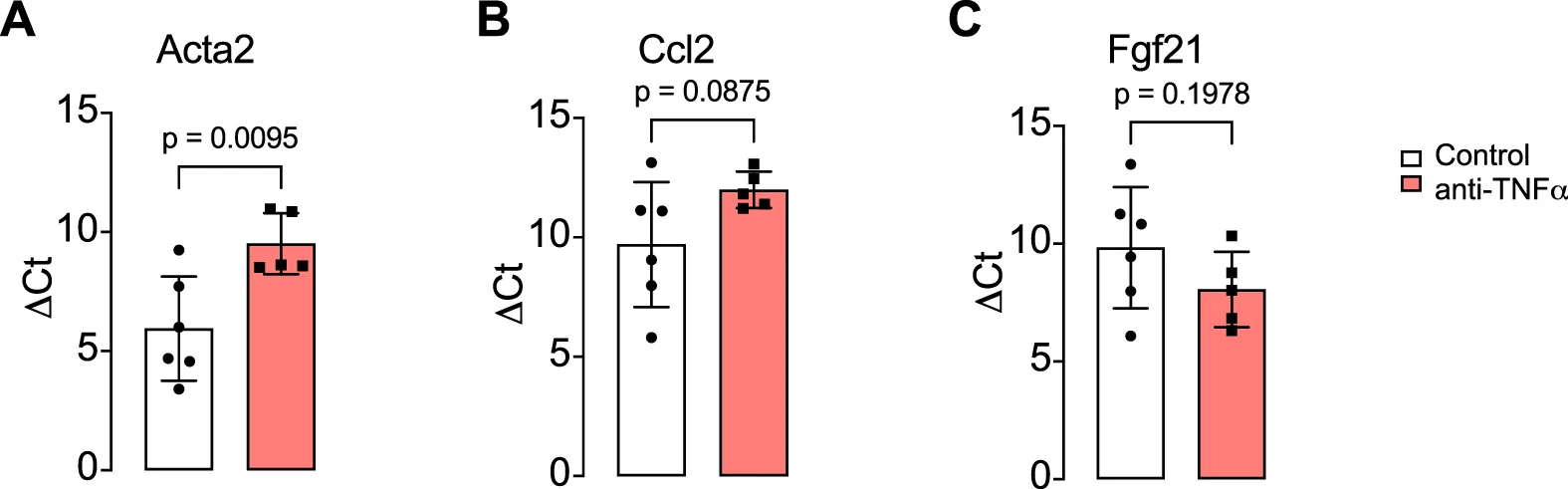
**Late TNFα blockade induces hepatic transcriptional remodeling at week 18.** Hepatic mRNA expression of (A) Acta2, (B) Ccl2 and (C) Fgf21 in emAIH mice treated with *anti*-TNFα or isotype control at week 18. Gene expression was quantified by quantitative real-time PCR and normalized to Actb and Gapdh. Data are shown as ΔCt values; higher ΔCt values indicate lower target gene expression. Each dot represents one animal; horizontal bars indicate the median. Statistics: Mann–Whitney *U* test, two-tailed. Exact p values are shown.

### 3.3 Late TNFα blockade induces compartment-specific T-cell rewiring

We next assessed whether the absence of biochemical improvement and reduced hepatic inflammatory infiltrate area was accompanied by changes in effector T-cell cytokine production. At week 18, the frequencies of IFN-γ-producing and TNFα-producing CD4^+^ and CD8^+^ T cells in spleen and liver were largely comparable between anti-TNFα-treated and isotype-treated mice (Supplementary Fig. S1A–D). A directional increase in splenic IFN-γ-producing CD4^+^ T cells was observed but did not reach statistical significance (Supplementary Fig. S1A).

By contrast, at week 24, anti-TNFα treatment was associated with increased frequencies of IFN-γ-producing and TNFα-producing splenic CD4^+^ T cells (Fig. 3A and B). Frequencies of cytokine-producing splenic CD8^+^ T cells showed no significant increase, although TNFα-producing CD8^+^ T cells showed a directional trend (Fig. 3A and B). This effect was compartment-specific: frequencies of IFN-γ-producing and TNFα-producing CD4^+^ and CD8^+^ T cells in the liver were not significantly altered by anti-TNFα treatment (Fig. 3C and D). Thus, the dominant treatment-associated cellular immune phenotype was observed systemically in the splenic CD4^+^ T-cell compartment rather than within the target organ.

**Fig. 3:**
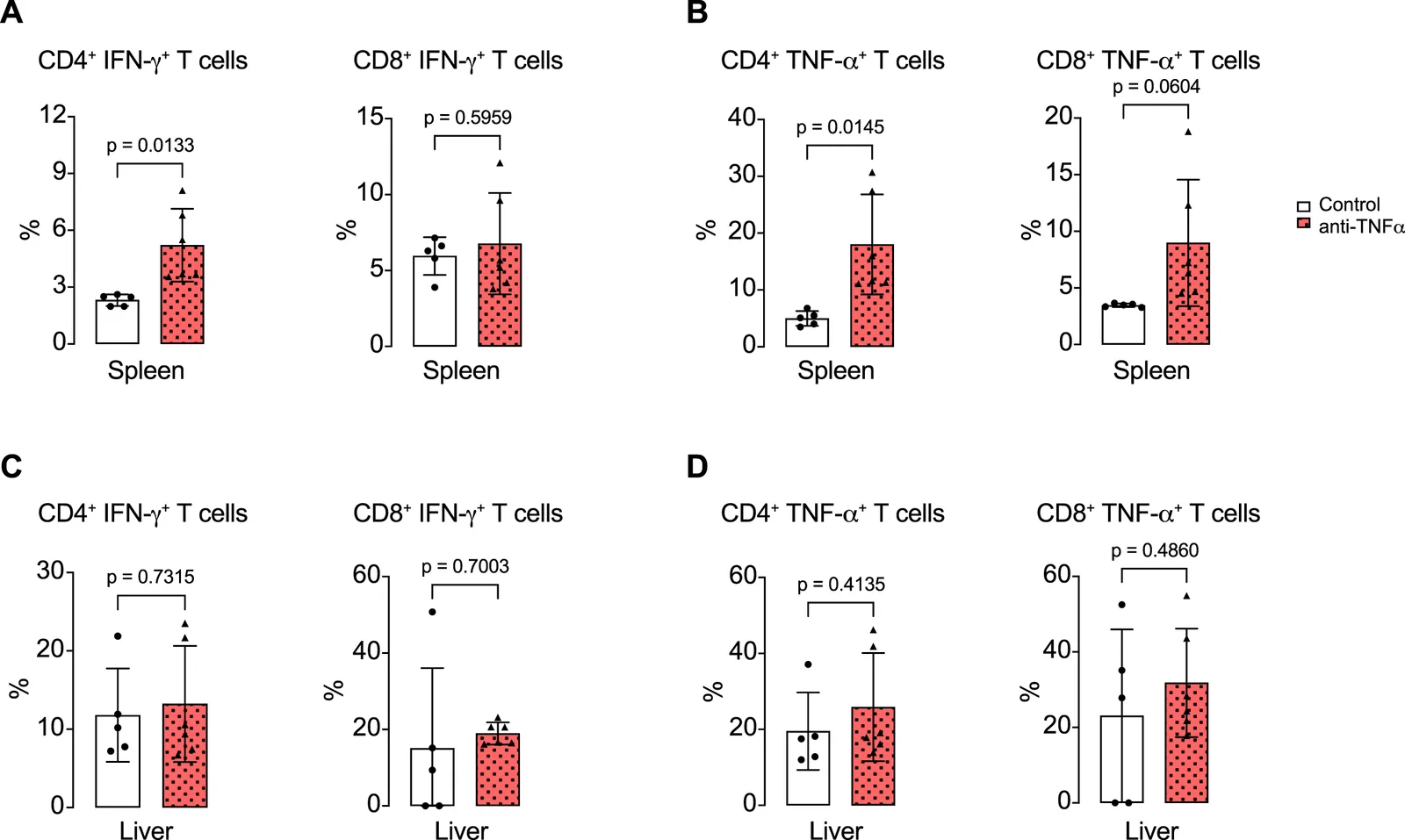
**Late TNFα blockade induces systemic CD4^+^ effector cytokine responses at week 24.** Flow cytometric analysis of intracellular IFN-γ and TNFα production by splenic and hepatic T cells at week 24 following TNFα blockade in established emAIH. (A) Frequencies of IFN-γ-producing CD4^+^ and CD8^+^ T cells in the spleen. (B) Frequencies of TNFα-producing CD4^+^ and CD8^+^ T cells in the spleen. (C) Frequencies of IFN-γ-producing CD4^+^ and CD8^+^ T cells in the liver. (D) Frequencies of TNFα-producing CD4^+^ and CD8^+^ T cells in the liver. Data are shown as percentages of the indicated T-cell subset. Each dot represents one animal; bars show the mean and standard deviation. Statistics: Mann–Whitney *U* test, two-tailed. Exact p values are shown.

### 3.4 Serum proteomics identifies late systemic inflammatory remodeling after TNFα blockade

To define systemic inflammatory changes associated with late TNFα blockade, serum proteomic profiling was performed using the Olink Mouse Inflammation panel at weeks 18 and 24. At week 18, anti-TNFα treatment was associated with only a limited serum protein signature, consistent with the modest cellular immune changes observed at this time point (Fig. 4A).

**Fig. 4:**
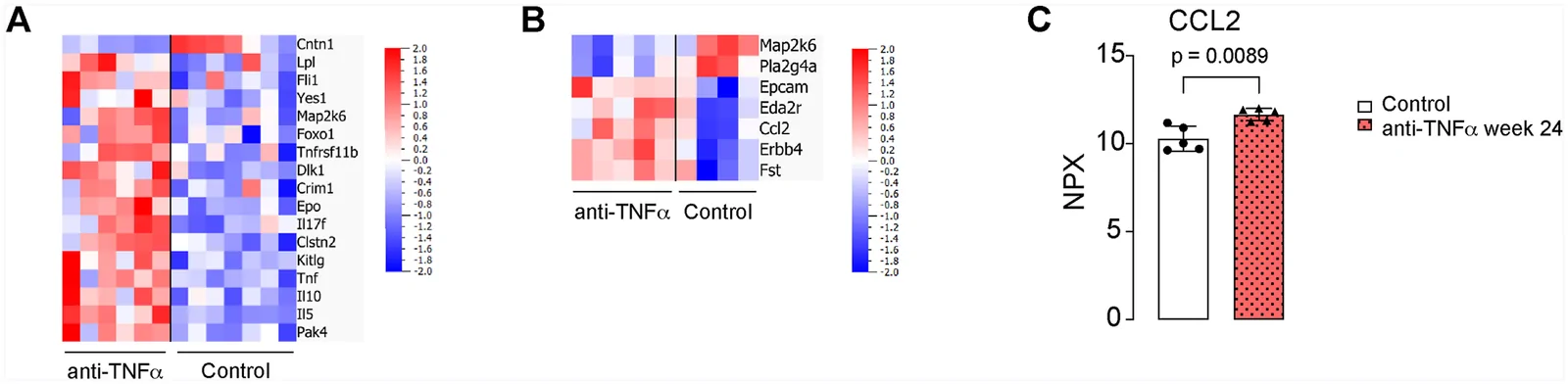
**Serum proteomics identifies late systemic inflammatory remodeling after TNFα blockade.** Serum inflammatory protein profiles were analyzed using the Olink Mouse Inflammation proximity extension assay panel at weeks 18 and 24 after AdFTCD administration. (A) Heatmap of serum protein abundance at week 18 comparing anti-TNFα-treated and isotype-treated emAIH mice. (B) Heatmap of serum protein abundance at week 24 comparing anti-TNFα-treated and isotype-treated emAIH mice. (C) Serum CCL2 abundance at week 24 shown as normalized protein expression (NPX) values. Heatmaps show row-scaled NPX values for visualization. Each column represents one animal. Protein abundance is reported on a log2 NPX scale, where higher values indicate higher protein abundance. Panel C shows individual animals with mean and standard deviation. Statistics: Mann–Whitney *U* test, two-tailed, or Olink differential abundance analysis as indicated. Exact p value is shown.

At week 24, serum proteomics revealed a more pronounced inflammatory remodeling pattern (Fig. 4B). In particular, CCL2 abundance was increased in anti-TNFα-treated mice compared with isotype-treated controls (Fig. 4C). This late CCL2 increase paralleled the emergence of increased splenic CD4^+^ effector cytokine frequencies and is consistent with treatment-associated systemic inflammatory remodeling despite the absence of improved serum transaminases or reduced hepatic inflammatory infiltration.

### 3.5 Late TNFα blockade does not durably reinforce regulatory immune signatures

Because TNF signaling can influence Treg biology, we next examined whether late TNFα blockade altered regulatory immune markers. Hepatic Foxp3 mRNA expression was not significantly increased at week 18 or week 24 after anti-TNFα treatment (Fig. 5A and B). At week 24, Foxp3 expression was numerically lower in anti-TNFα-treated mice, but this did not reach statistical significance (Fig. 5B).

**Fig. 5:**
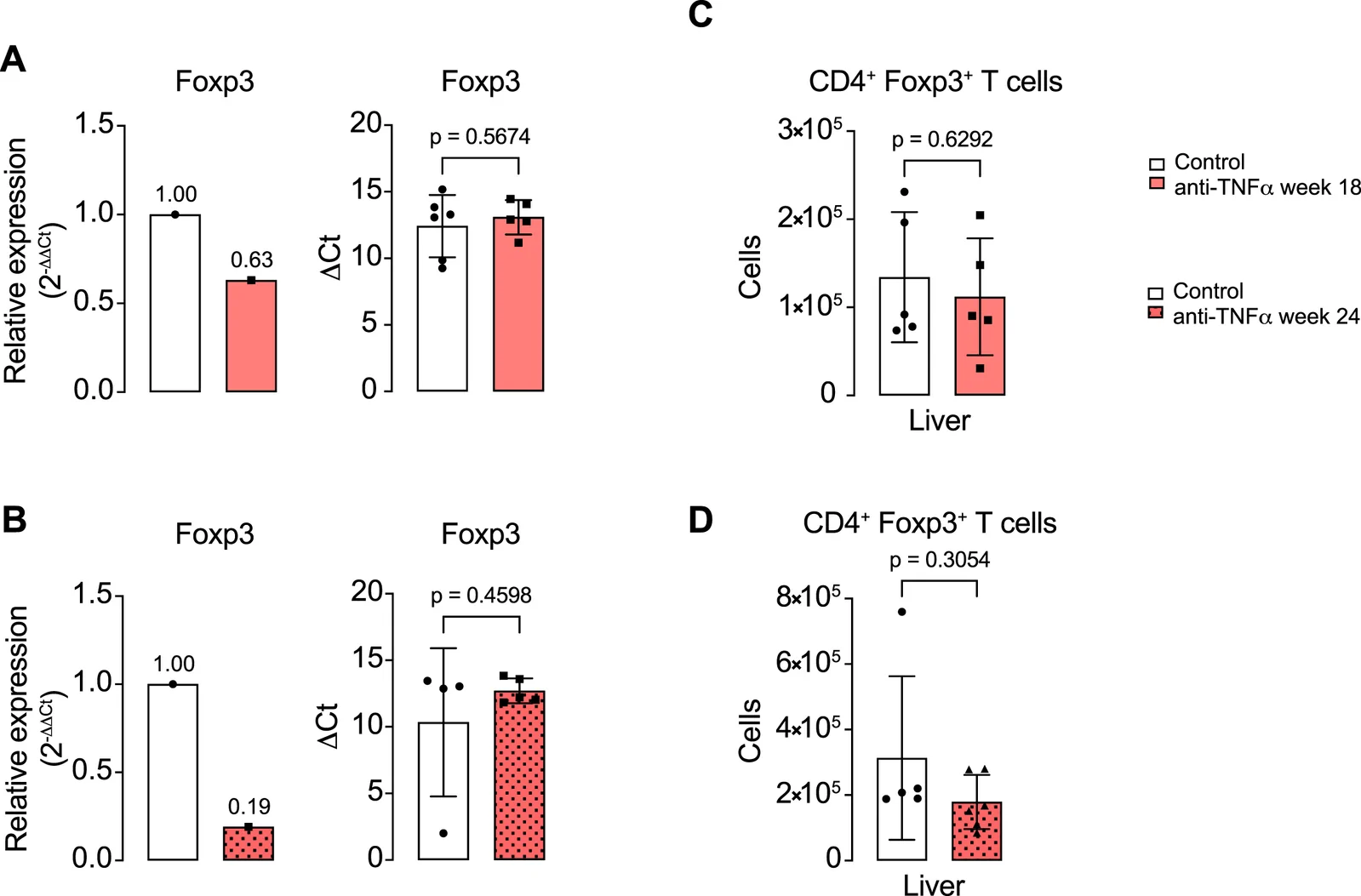
**Late TNFα blockade does not durably reinforce hepatic regulatory immune signatures.** Regulatory immune readouts were assessed at weeks 18 and 24 following late TNFα blockade in established emAIH. (A) Hepatic Foxp3 mRNA expression at week 18. (B) Hepatic Foxp3 mRNA expression at week 24. (C) Absolute numbers of hepatic CD4^+^Foxp3^+^ regulatory T cells at week 18. (D) Absolute numbers of hepatic CD4^+^Foxp3^+^ regulatory T cells at week 24. Foxp3 expression was quantified by quantitative real-time PCR and normalized to Actb and Gapdh. ΔCt values are shown for statistical comparison; higher ΔCt values indicate lower Foxp3 expression. CD4^+^Foxp3^+^ regulatory T cells were quantified by flow cytometry. Each dot represents one animal; bars indicate the mean and standard deviation. Statistics: Mann–Whitney *U* test, two-tailed. Exact p values are shown.

Consistently, the absolute numbers of hepatic CD4^+^CD25^+^Foxp3^+^ Tregs were not increased by anti-TNFα treatment at week 18 or week 24 (Fig. 5C and D). Extended analyses of CD62L and Ki67 expression did not indicate broad changes in T-cell activation or proliferation across hepatic and splenic CD4^+^ or CD8^+^ T-cell compartments (Supplementary Fig. S2A–D). Additional Treg analyses likewise did not support sustained proliferative reinforcement of the regulatory compartment following TNFα blockade (Supplementary Fig. S3A–C). Thus, late TNFα blockade was associated with systemic CD4^+^ effector cytokine remodeling without durable reinforcement of hepatic regulatory immune signatures.

## 4 Discussion

Our study addresses a biologically distinct question from the recent human infliximab studies in AIH and should therefore be interpreted as complementary rather than contradictory. While Xu et al. identified TNF as a pathogenic node in newly diagnosed AIH and demonstrated steroid-free clinical efficacy of infliximab in early active disease [8], and Efe et al. reported biochemical benefit in a subset of difficult-to-treat patients treated in real-world rescue settings [7], our experimental work specifically interrogates the consequences of TNFα blockade initiated after disease establishment in chronic autoimmune liver inflammation. Thus, the present study does not challenge the therapeutic relevance of TNFα in AIH, but defines a different disease context in which TNFα neutralization may have distinct biological consequences.

In this setting, anti-TNFα treatment was associated with early hepatic molecular changes but failed to improve serum transaminases or reduce hepatic inflammatory infiltrate area. This central dissociation shaped the subsequent immune phenotype: late anti-TNFα treatment was associated with time point- and compartment-dependent immune phenotype, with increased frequencies of IFN-γ- and TNFα-producing splenic CD4^+^ T cells, whereas intrahepatic cytokine-producing T-cell frequencies remained largely unchanged. The key finding is therefore not a simple absence of anti-inflammatory activity, but the coexistence of measurable molecular and systemic changes with no biochemical improvement or reduction in hepatic inflammatory infiltrate area.

This distinction is clinically relevant because anti-TNF therapy is generally considered only in patients who are intolerant of, incompletely responsive to, or refractory to standard first-line immunosuppression, that is, in the very rescue setting in which biological mechanisms of response versus non-response are most urgently needed [[4], [5], [6], [7]]. Incomplete biochemical response is a major determinant of adverse long-term outcome in AIH [20,21], which makes the absence of transaminase improvement in our model a meaningful finding rather than a negative technical result. In parallel, unchanged hepatic inflammatory infiltrate area indicates that the lack of biochemical improvement was not accompanied by an obvious reduction in inflammatory tissue infiltration. Our data suggest that, once autoimmune liver inflammation is established, TNFα neutralization may become uncoupled from biochemical improvement and reduced inflammatory burden while still being associated with systemic effector immune changes. One possible explanation for the divergence from the clinical benefit observed in selected human infliximab studies [7,8] is that the relative dependence of autoimmune inflammation on TNFα may change with disease timing and context. Newly diagnosed active AIH may contain a more TNFα-dependent inflammatory network, whereas established chronic inflammation may increasingly be sustained by redundant effector pathways, persistent antigen-driven responses, tissue remodeling, and altered regulatory-effector interactions. Under these conditions, TNFα neutralization may remain biologically active without being sufficient to improve biochemical activity or reduce inflammatory infiltrate burden.

Mechanistically, this is plausible in light of the context-dependent functions of TNFα. Beyond its classical inflammatory role [9], TNF signaling can contribute to T-cell homeostasis, Treg stability and suppressive function via TNFR2-dependent pathways [10,11]. Thus, TNFα neutralization in chronic disease may not simply “switch off” inflammation but may perturb the balance between regulatory and effector compartments in a context-dependent manner. This interpretation is supported by the temporal dissociation observed in our study: hepatic transcriptional modulation was detected at week 18, whereas the most prominent cellular and proteomic changes emerged systemically at week 24. The splenic-liver discrepancy may reflect differences in the stability and regulation of peripheral versus established tissue inflammatory compartments. Persistent local antigen presentation, tissue-associated immune-cell retention, and redundant inflammatory signals may render the intrahepatic compartment less responsive to perturbation of a single cytokine pathway, whereas peripheral lymphoid populations may remain more dynamically responsive to altered cytokine availability, activation, survival, or trafficking. Conversely, the increased frequencies of cytokine-producing splenic CD4^+^ T cells could reflect compensatory immune remodeling, paradoxical immune activation, or altered peripheral homeostasis rather than a direct activating effect of TNFα blockade. Because cellular trafficking, tissue residency, clonality, and thymic output were not directly assessed, these possibilities remain mechanistic hypotheses rather than conclusions from the present data.

Our data are also consistent with the broader heterogeneity of immune regulation reported in AIH. Human studies have documented altered Treg-associated phenotypes and suppressive capacity [12], treatment-associated depletion of intrahepatic Tregs under conventional immunosuppression [13], and preservation or inflammation-associated accumulation of FOXP3^+^ cells in other cohorts [14]. In our model, late anti-TNFα therapy did not durably reinforce regulatory signatures: Foxp3 expression was not increased, and absolute hepatic Treg numbers were not increased. This places TNF blockade in contrast to experimental interventions that directly reinforce immune regulation, such as IL-2–based Treg support [19], and aligns more closely with other pathway-directed interventions in emAIH that alter molecular signatures without necessarily restoring biochemical control, such as anti-CD20 treatment [22]. Together, these observations support the concept that successful treatment of established autoimmune liver inflammation may require restoration of durable immune regulation rather than isolated cytokine neutralization.

The serum proteomic findings further support this interpretation but should be considered exploratory. Increased CCL2 abundance at the late time point is consistent with persistent or reorganized inflammatory signaling rather than resolution. CCL2/MCP-1 is a key chemokine involved in monocyte/macrophage migration and inflammatory cell infiltration; therefore, its increase despite TNFα blockade is compatible with persistent chemokine-driven inflammatory activity rather than complete immunological resolution [23]. However, given the small cohort size and high-dimensional nature of the Olink analysis, individual protein changes should not be overinterpreted in isolation. Their relevance lies primarily in their concordance with the late systemic CD4^+^ effector cytokine phenotype.

This study has limitations. It uses a single murine model, and differences between weeks 18 and 24 reflect both disease timing and treatment duration. Although contemporaneous isotype-treated controls account for general time-dependent evolution of emAIH, treatment-associated effects cannot be fully separated from interactions with progressive immune changes in chronic disease. Individual pretreatment biochemical and histological baselines were not available; because liver histology requires terminal tissue collection, disease establishment at week 12 relies on the previously validated kinetics of the AdFTCD/NOD/Ltj model. Moreover, our model does not reproduce the biochemical benefit observed in newly diagnosed human AIH under infliximab, which limits direct translational extrapolation. Histological assessment was restricted to quantitative measurement of inflammatory infiltrate area and did not distinguish portal from lobular inflammation, infiltrate composition, interface activity, or other components of parenchymal injury; our conclusions are therefore limited to inflammatory infiltrate burden rather than comprehensive histological disease activity. TNFα activity was not directly quantified as bioactive circulating or intrahepatic protein after antibody treatment; instead, TNF-related effects were inferred from hepatic transcriptional, cellular and proteomic readouts. T-cell trafficking, tissue residency, clonality, and thymic output were not directly assessed, limiting mechanistic interpretation of the splenic–hepatic discrepancy. Multiparameter immunophenotyping also entails multiple comparisons; accordingly, findings beyond the consistent splenic CD4 cytokine signal at week 24 should be considered exploratory. Finally, anti-TNF therapy is associated with clinically relevant infectious complications in rescue cohorts [7] and drug-induced liver injury in broader treated populations [24], which should be considered when extrapolating mechanistic observations to clinical application.

In conclusion, late anti-TNFα treatment in established emAIH is biologically active at the hepatic molecular level but does not improve biochemical disease activity or reduce hepatic inflammatory infiltration and is associated with time point- and compartment-dependent immune remodeling. Rather than arguing against the therapeutic relevance of TNFα in AIH, these data indicate that the biological consequences of TNFα-targeted therapy may depend on disease context and timing. They complement the clinical observations by Xu et al. and Efe et al. by suggesting that TNFα blockade may remain biologically active even when it does not translate into measurable disease control in established autoimmune liver inflammation. This supports further work on treatment timing, patient stratification and combination strategies that restore durable immune regulation.

## CRediT authorship contribution statement

**Laura Elisa Buitrago-Molina:** Writing – original draft, Visualization, Validation, Software, Methodology, Investigation, Funding acquisition, Formal analysis. **Zexuan Shi:** Writing – review & editing, Methodology, Investigation. **Ziting Zhao:** Writing – review & editing, Investigation. **Heiner Wedemeyer:** Writing – review & editing, Resources, Formal analysis. **Fatih Noyan:** Writing – review & editing, Resources, Formal analysis. **Matthias Hardtke-Wolenski:** Writing – review & editing, Writing – original draft, Supervision, Resources, Project administration, Funding acquisition, Formal analysis, Data curation, Conceptualization.

## Informed consent statement

Not applicable. This study did not involve human participants.

## Institutional animal care and use committee statement

All animal experiments were approved by the responsible institutional and governmental authorities and were conducted in accordance with national and institutional guidelines for the care and use of laboratory animals.

## Arrive guidelines statement

The authors have read the ARRIVE guidelines, and the manuscript was prepared and revised according to the ARRIVE guidelines.

## Data sharing statement

All relevant data are included in the manuscript and its supplementary materials. Additional data are available from the corresponding author upon reasonable request.

## Funding statement

This work was supported by grants from the 10.13039/501100001659German Research Foundation (BU 2722/2-3 and HA 6880/2-3).

## Declaration of competing interest

The authors declare that they have no known competing financial interests or personal relationships that could have appeared to influence the work reported in this paper.

## Data Availability

Data will be made available on request.
