# Supplementary material for: Late TNFα blockade is associated with systemic immune rewiring without disease control in established experimental autoimmune hepatitis
Source: J Transl Autoimmun. 2026 Aug 25;13:100396. doi: 10.1016/j.jtauto.2026.100396 (PMC13640463; doi:10.1016/j.jtauto.2026.100396)
Supplement: Supplementary file 1 [file mmc1.docx]

 **Supplementary Figure S1. Extended week-18 cytokine profiling after late TNFα blockade.**

Extended flow cytometric analysis of intracellular IFN-γ and TNFα production by CD4⁺ and CD8⁺ T cells in spleen and liver at week 18 following TNFα blockade in established emAIH. (A) Frequencies of IFN-γ-producing CD4⁺ and CD8⁺ T cells in the spleen. (B) Frequencies of TNFα-producing CD4⁺ and CD8⁺ T cells in the spleen. (C) Frequencies of IFN-γ-producing CD4⁺ and CD8⁺ T cells in the liver. (D) Frequencies of TNFα-producing CD4⁺ and CD8⁺ T cells in the liver. Data are shown as percentages of the indicated T-cell subset. Each dot represents one animal; bars show the mean and standard deviation. Statistics: Mann–Whitney U test, two-tailed. Exact p values are shown.

**Supplementary Figure S2. T-cell activation and proliferation markers after late TNFα blockade.**

Flow cytometric analysis of CD62L and Ki67 expression in hepatic and splenic T cells at weeks 18 and 24 following late TNFα blockade in established emAIH. (A) Frequencies of CD62L⁺ and Ki67⁺ CD4⁺ T cells in the liver. (B) Frequencies of CD62L⁺ and Ki67⁺ CD8⁺ T cells in the liver. (C) Frequencies of CD62L⁺ and Ki67⁺ CD4⁺ T cells in the spleen. (D) Frequencies of CD62L⁺ and Ki67⁺ CD8⁺ T cells in the spleen. Data are shown as percentages of the indicated T-cell subset. Each dot represents one animal; bars show the mean and standard deviation. Statistics: Mann–Whitney U test, two-tailed. Exact p values are shown.

 **Supplementary Figure S3. Extended regulatory T-cell proliferation analyses after late TNFα blockade.**

Extended flow cytometric analysis of regulatory T-cell frequencies and proliferation after late TNFα blockade in established emAIH. (A) Frequencies of splenic CD4⁺Foxp3⁺ regulatory T cells at week 18. (B) Frequencies of Ki67⁺ cells among hepatic CD4⁺Foxp3⁺ regulatory T cells at weeks 18 and 24. (C) Frequencies of Ki67⁺ cells among splenic CD4⁺Foxp3⁺ regulatory T cells at weeks 18 and 24. Data are shown as percentages of the indicated T-cell subset. Each dot represents one animal; bars show the mean and standard deviation. Statistics: Mann–Whitney U test, two-tailed. Exact p values are shown.
